# Sodium Intake and Related Diseases

**DOI:** 10.3390/ijms22147608

**Published:** 2021-07-16

**Authors:** Massimo Lucarini, Alessandra Durazzo, Stefania Sette, Ginevra Lombardi-Boccia, Antonello Santini, Pasquale Strazzullo

**Affiliations:** 1 CREA-Research Centre for Food and Nutrition, Via Ardeatina 546, 00178 Rome, Italy; alessandra.durazzo@crea.gov.it (A.D.); stefania.sette@crea.gov.it (S.S.); g.lombardiboccia@crea.gov.it (G.L.-B.); 2 Department of Pharmacy, University of Napoli Federico II, Via D. Montesano 49, 80131 Napoli, Italy; asantini@unina.it; 3 Department of Clinical Medicine and Surgery, Federico II University of Naples, Via S. Pansini 5, 80131 Naples, Italy

Moderation in the use of salt (sodium chloride) in food and food preparations prevents the tendency of blood pressure to increase with age, and this is documented by many studies in current literature. In contrast, the abuse of salt frequently leads to increases in blood pressure and contributes to the development of hypertension, particularly in overweight or obese people, in diabetics, in the elderly and in genetically predisposed subjects.

Given the well-known relationship between high blood pressure and the risk of cardiovascular disease, high salt consumption is also associated with an increased risk of fatal or otherwise debilitating cardiovascular events, with a high impact on health expenditure. 

The reduction of salt consumption leads to a decrease in blood pressure—more so in hypertensive, elderly and obese subjects—and consequently to a reduction in cardiovascular risk. The development of salt-reducing programs for individual, population, and country-level strategies to reduce salt intake is becoming a challenge, considering the general tendency not to change the use of salt. Improving knowledge is a key step for behavioral changes, suggesting the need for effective public health interventions throughout educational campaigns addressed at the implementation of good practices in nutrition [1,2].

The nutritional goal for the adult population has been set at no more than 2000 mg of sodium or 5 g of salt per day, in keeping with the WHO recommendation [3] that applies to all adult individuals, including the elderly, in the absence of different medical/nutritional indications.

At least half of the amount of salt taken individually comes from processed foods and food purchased and/or consumed outside of the home, and for these reasons an effective reduction in salt consumption requires the active participation of the food industry and the awareness of the consumer to the sodium content of the consumed products.

This Special Issue is focused on the role of sodium in the body’s physiological processes. Generally, complex mechanisms regulate sodium concentrations in bodily fluids that involve the cardiovascular and endocrine systems, the central nervous system and the autonomic nervous system. The mechanisms involved in the regulation of sodium homeostasis will be the focus of this Special Issue. Some examples are the mechanisms which influence the action of the sodium–potassium pump, the renal tubular reabsorption mechanisms regulated by hormones, such as angiotensin II, and norepinephrine and those of sodium elimination, regulated by dopamine and cyclic AMP. The mechanisms involved at the molecular level of the relationship between sodium intake–blood pressure–cardiovascular disease and stomach cancer are among the focuses of this Special Issue.

The main topics of this Special Issue include: levels of intake and main sources of sodium from the diet: effect on the health status and description of the biochemical processes involved; salt intake and related risks; studies in the management and treatment of sodium intake-related diseases; epidemiological studies of the relationship between salt intake and related diseases: focus on the mechanism of action; delineation of mechanism of actions: *in vitro* and *in vivo* studies; salt and sapidity: mechanisms of taste perception. Chan et al. [4] investigated the MST3 involvement in Na^+^ and K^+^ homeostasis with increasing dietary potassium intake, in mice fed by diets containing various concentrations of Na^+^ and K^+^. The 2% KCl diets induced less MST3 expression in MST3^−/−^ mice than that in wild-type (WT) mice. The MST3^−/−^ mice had higher WNK4, NKCC2-S130 phosphorylation, and ENaC (epithelial Na channel) expression, resulting in lower urinary Na^+^ and K^+^ excretion than those of WT mice. Lower urinary Na^+^ excretion was associated with elevated plasma [Na^+^] and hypertension. The authors marked how MST3 maintains Na^+^/K^+^ homeostasis in response to K^+^ loading by regulation of WNK4 expression and NKCC2 and ENaC activity [4].

Hirohama et al. [5] showed, using animal models, how PGI2 analog attenuates salt-induced renal injury through the inhibition of inflammation and Rac1-MR activation; this study clearly demonstrated that Beraprost sodium (BPS), a pharmaceutical used in several Asian countries, including Japan and South Korea, as a vasodilator and antiplatelet agent, had renoprotective effects in salt-induced kidney injury, leading to the plausible hypothesis that BPS is therapeutically useful for the treatment of salt-induced renal damage.

Nakayama et al. [6] reported how Na^+^ coupled nutrient cotransport-induced luminal negative potential and claudin-15 play an important role in paracellular Na^+^ recycling in mouse small intestine; particularly, the authors concluded that Na^+^, which is absorbed by Na^+^-dependent glucose cotransport, is recycled back into the lumen via paracellular Na^+^ conductance through claudin-15, which is driven by Na^+^ cotransport induced luminal negativity [6].

It is worth mentioning the reviews by Patel and Joseph [7] on sodium intake and heart failure as well as that by Borrelli et al. [8] on sodium intake and chronic kidney disease.

This Special Issue contributes to the field of research on sodium, aiming to better understand its mechanism of action and reference and the relationship between sodium intake and related diseases.
